# Trends in robot-assisted and virtual reality-assisted neuromuscular therapy: a systematic review of health-related multiplayer games

**DOI:** 10.1186/s12984-018-0449-9

**Published:** 2018-11-19

**Authors:** Kilian Baur, Alexandra Schättin, Eling D. de Bruin, Robert Riener, Jaime E. Duarte, Peter Wolf

**Affiliations:** 1 Sensory-Motor Systems Lab, Department of Health Sciences and Technology, ETH Zurich, Tannenstrasse 1, Zurich, 8092 Switzerland; 2 Spinal Cord Injury Center, University Hospital Balgrist, University of Zurich, Zurich, Switzerland, Forchstrasse 340, Zurich, 8008 Switzerland; 3 Department of Health Sciences and Technology, Institute of Human Movement Sciences and Sport, Swiss Federal Institute of Technology (ETH Zurich), Zurich, Switzerland, Leopold-Ruzicka-Weg 4, Zurich, 8093 Switzerland; 4 Department of Neurobiology, Care Sciences and Society, Karolinska Institutet, Stockholm, Sweden, Alfred Nobels Alle 23, Huddinge, 141 83 Sweden; 5 MyoSwiss AG, Lengghalde 5, Zürich, CH-8008 Switzerland

**Keywords:** Neurorehabilitation, Multiplayer, Flow, Challenge, Robotic assistance

## Abstract

**Background:**

Multiplayer games have emerged as a promising approach to increase the motivation of patients involved in rehabilitation therapy. In this systematic review, we evaluated recent publications in health-related multiplayer games that involved patients with cognitive and/or motor impairments. The aim was to investigate the effect of multiplayer gaming on game experience and game performance in healthy and non-healthy populations in comparison to individual game play. We further discuss the publications within the context of the theory of flow and the challenge point framework.

**Methods:**

A systematic search was conducted through EMBASE, Medline, PubMed, Cochrane, CINAHL and PsycINFO. The search was complemented by recent publications in robot-assisted multiplayer neurorehabilitation. The search was restricted to robot-assisted or virtual reality-based training.

**Results:**

Thirteen articles met the inclusion criteria. Multiplayer modes used in health-related multiplayer games were: competitive, collaborative and co-active multiplayer modes. Multiplayer modes positively affected game experience in nine studies and game performance in six studies. Two articles reported increased game performance in single-player mode when compared to multiplayer mode.

**Conclusions:**

The multiplayer modes of training reviewed improved game experience and game performance compared to single-player modes. However, the methods reviewed were quite heterogeneous and not exhaustive. One important take-away is that adaptation of the game conditions can individualize the difficulty of a game to a player’s skill level in competitive multiplayer games. Robotic assistance and virtual reality can enhance individualization by, for example, adapting the haptic conditions, e.g. by increasing haptic support or by providing haptic resistance. The flow theory and the challenge point framework support these results and are used in this review to frame the idea of adapting players’ game conditions.

**Electronic supplementary material:**

The online version of this article (10.1186/s12984-018-0449-9) contains supplementary material, which is available to authorized users.

## Introduction

### Robotic assistance and virtual reality in neuromuscular therapy

Neurological deficits can result in impaired motor function that affect a person’s quality of life. Researchers have been working to restore the nervous system and reduce the neurological deficits of people suffering from stroke, spinal cord injury, or traumatic brain injury [1]. For people with neurological deficits, impaired motor function is among the most prominent factors limiting the quality of life [2]. Motor neurorehabilitation can lead to permanent improvements in motor function [3]. Robotic assistance and virtual reality have the potential to enhance rehabilitation of neuromuscular deficits beyond the levels possible with conventional training strategies [4, 5].

### Game experience and task performance in multiplayer games

Robot- and virtual reality-assisted single-player games are well integrated in neurorehabilitation schedules. Recently, multiplayer games have been tested to complement neuromuscular therapy. Multiplayer games are expected to motivate the patients and increase the potential of robot- and virtual reality-assisted neuromuscular therapy.

Multiplayer games incorporate social interaction to promote the enjoyment of the involved players. The additional player adds new possibilities to the game environment, generally missed in single-player gaming against preprogrammed challenges or artificially controlled opponents. The multiplayer environment and related game mechanics can facilitate social interaction, ranging from conversation to haptic interaction. Due to the this added social interaction, the game experience is thought to be better in multiplayer compared to single-player gaming [6].

The mode of the game specifies whether the players compete or cooperate with one another [7]. In line with the flow theory, a competitive mode requires opponents of similar skill level to achieve enjoyment as the task difficulty experienced by one opponent [8]. Comparable skill levels prevent boredom or stress and result in a meaningful challenge level that leads to a flow state when training [9]. In such training conditions the players have a positive game experience.

In positive game experience players increase their game performance [9, 10]. Increased game performance facilitates the general idea of serious games, i.e., playing for a primary purpose other than pure entertainment [11]. If enhanced game performance is achieved by increased physical activity, training intensity is also increased. In neuromuscular therapy, training intensity – alongside early treatment, user-centered, and task-oriented training – is one of the key factors in neurorehabilitation [12, 13]. Therefore, multiplayer gaming has great potential to further increase the benefits of robot-assisted neuromuscular and virtual reality-assisted therapy [14, 15].

### Differently skilled patients and motor learning

Accounting for the individual’s skill level is not only important for game experience, but also for motor learning. According to the challenge point framework [16], motor learning depends on the amount of interpretable information. An increase in task difficulty can increase the amount of available information. However, the learning potential is only increased when information can still be interpreted and does not overload the individual, i.e. hamper task performance. As the ability to process information varies between individuals, the task difficulty should be adjusted to the individual in order to facilitate learning.

### Manipulation of game conditions in multiplayer games

Various solutions have been offered to account for the differences in skill level in multiplayer gaming in neurorehabilitation. These can be differentiated into adaptation of game mechanics (e.g. frequency of actions) and adaptation of the interface’s mechanics (e.g. robotic support). In multiplayer games, adaptations of the game mechanics affect the game condition for all players. In contrast, adaptation at the interface level allows for individual adjustment of the task difficulty. Thus, in settings with differently skilled players, individual interface mechanics can ensure that all players are challenged according to their skill level and can therefore increase game experience.

In health-related gaming, task difficulty adaptation in multiplayer games, i.e. accounting for the skill level of the opponents, bears a challenge for game developers: Adaptations of game mechanics–as commonly done for single-player games–affect the game conditions for all players. In health-related gaming environments – particularly in patients with neuromuscular deficits – this challenge is even more prominent due to large variability in information processing abilities and motor skills. This large variability leads to differences across active range of motion, muscular strength, interlimb coordination or spasticity, among others. In multiplayer games, these deficits can make it so that two players cannot play against one another. It is therefore of interest to understand how to manipulate game conditions to balance the skill levels of patients and enable multiplayer gaming.

### Contribution of this review

In this review, we investigated whether multiplayer environments have improved game experience or game performance in serious games for health-related disciplines and neuromuscular therapy. We further compared different multiplayer game modes regarding game experience and game performance. To facilitate the transfer and consolidation of multiplayer gaming from health-related gaming to robot-assisted neuromuscular therapy, we systematically reviewed the literature regarding multiplayer gaming in health-related games. In this article, we describe the available literature in the context of the flow model and the challenge point framework. To further facilitate a transfer to neuromuscular rehabilitation, this review discusses measures regarding perceived game experience and physical performance, which have been applied in studies using multiplayer modes offering social interaction. Furthermore, this review complements definitions of task difficulty with the term “conditional task difficulty” to facilitate the discussion of haptic difficulty adaptation in robot-assisted rehabilitation. All discussions refer to our research question: Do multiplayer games enhance experience and performance in robot-assisted and virtual-reality assisted neurorehabilitation?

## Methods

### Protocol

The review protocol was based on an initial search review of the promising aspects of social interaction in virtual environments for rehabilitation interventions.

### Eligibility criteria

We developed a database adjusted electronic search strategy for EMBASE, Medline, PubMed, Cochrane, CINAHL and PsycINFO in collaboration with a librarian from the Medicinal Library of the University of Zurich. The search was restricted to English and German language literature. There was no limitation in publication date or restriction by study design. The first search was performed in April 2016 and was complemented by papers published since (until January 2018).

We used medical sub-headings as search terms, including the following main terms for the population: motor impairments, cognitive impairments, stroke, elderly people; for interventions: competition, collaboration, cooperation, coopetition, competitive behavior, collaborative behavior, exergame, multi-player-exergame, serious game, rehabilitative game, rehabilitative exergame, education game, computerized training, robot-assisted rehabilitation, robot-aided rehabilitation, virtual reality, virtual reality therapy, virtual world, social community, community integration, virtual community; for outcome: persuasion, compliance, motivation, engagement, effort, adherence, therapy progress.

The search strategy (Additional file 1) was initially run in EMBASE and then adapted to the search format requirements of the other databases included in this review. The search results were supplemented by articles found through manual searches by scanning reference lists of identified studies.

### Study selection

After duplicate citations were removed, two independent reviewers (KB, AS) determined which articles should be included within this systematic review by scanning the titles, abstracts and keywords while applying the inclusion and exclusion criteria (Table 1). An article was included if both reviewers independently saw the potential that the article’s results might be of interest for multiplayer applications in robot-assisted neuromuscular therapy. To determine whether the full text should be retrieved for a given citation, the two reviewers marked each citation using a “yes,” “no,” or “unknown” (unsure whether yes or no) designation. The citations marked as “unknown” were discussed within the reviewers. The screening process involved simultaneous title and abstract screening. The reviewers evaluated the same set of citations. A study was considered eligible for inclusion in the review when the studied intervention: (1) was a multiplayer intervention in a robot-assisted, video-game based or virtual reality based, health-related setting, and (2) examined the effects of the intervention on perceived game experience or physical functioning of the players involved.

**Table 1:** List of inclusion and exclusion details

Area	Inclusion details
Population	Healthy, obesity, motor impairments, cognitive impairments, stroke, elderly people
Study type	Intervention studies of any type, including case studies and non-randomized trials
Intervention	Competition, collaboration, cooperation, coopetition, competitive behavior, collaborative behavior, exergame, multi-player-exergame, serious game, rehabilitative game/exergame, education game, computerized training, robot-assisted rehabilitation, robot-aided rehabilitation, virtual reality, virtual reality therapy, virtual world, social community, community integration, virtual community, video-game based
Outcomes	Persuasion, compliance, motivation, engagement, effort, adherence, therapy progress, therapy success
	Exclusion details
	Reviews, animal studies, concept studies, feasibility studies, human-machine interaction, methodological theoretical or discussion papers

This review includes a transfer to neurorehabilitation training gaming from related disciplines, such as health related serious gaming. For this purpose, we considered typical types of exercise interfaces as described by Tanaka [17]. Feasibility studies with less than ten subjects were not included in the systematic assessment but relevant content was considered for the discussion.

**Fig. 1:**
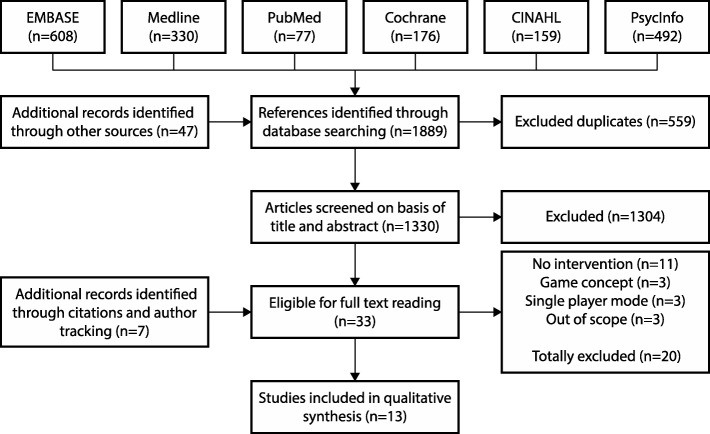
Study selection flow chart

The full-text article was read if the title, abstract, or keywords provided insufficient information to decide on inclusion (Fig. 1).

### Data extraction process and data synthesis

The following data were extracted from the studies: (1) characteristics of the studied population: number of participants, disease and age, (2) characteristics of the interventions: the design, frequency and duration of the intervention, co-interventions and control intervention; (3) characteristics of the outcomes: outcome measures and results (Tables 2, 3, 4, and 5). The included studies were divided into three groups according to their assessments: perceived game experience (Table 3), physical performance (Table 4), and personality factors (Table 5). The review of perceived game experience included studies using inventories that assessed the players’ game experience or game playtime expressing a positive game experience. The review of physical performance included studies assessing any physical fitness related quantity measured by the game score or physical quantities measured on the player. If a study monitored both perceived game experience and physical performance, the study appears in every relevant group, accordingly.

**Table 2:** Included studies reported by design and subject specifications

Study	Design	N	Subjects	Age range or mean age ± one standard deviation (years)
Feltz et al. 2012 [34]	Randomized	135	Undergraduate students	19.8±2.7
Ganesh et al. 2014 [27]	Within subject	74	Healthy subjects	25 …42
Goršič et al. 2017 [31]	Within subject	29	Patients (ischemic stroke: 19; hemorrhagic stroke: 3; brain tumor: 4; shoulder rotator cuff tear: 2; traumatic brain injury: 1) with chronic arm impairment	56.7±14.7
Goršič et al. 2017 [32]	Within subject	35	15 patients (ischemic stroke: 9; hemorrhagic stroke: 1; ischemic stroke followed by hemorrhagic stroke: 2; traumatic brain injury: 1; cerebral palsy 2) with chronic arm impairment / 20 patients in the acute or subacute phase of stroke	52.7±13.7/57.8±11.7
Goršič et al. 2017 [33]	Within subject	69	64 unimpaired friends (undergraduate students) / 5 arm impaired due to neurological injuries (ischemic stroke: 3; traumatic brain injury: 1; spinal cord injury: 1)	25.6±6.9/24…51
Johnson et al. 2008 [28]	Within subject	18	Healthy subjects	21 …62
Mace et al. 2017 [29]	Within subject	48	32 healthy / 16 hemiparetic stroke survivors using their impaired arm	26.3±4.5/70.3±19.7
Novak et al. 2014 [30]	Within subject	38	30 unimpaired (no cognitive or motor impairment) / 8 stroke patients	25 …73 / 22 …69
Peng and Crouse 2013 [35]	Assigned	162	Undergraduate communication class	18 …23
Peng and Hsieh 2012 [36]	Within subject	158	Undergraduate communication class at a large Midwestern university	18 …23
Staiano et al. 2012 [25]	Randomized	31	Low-income, adolescents, public high school	15 …19
Staiano et al. 2013 [26]	RCT	31	Adolescents, urban public high school	15 …19
Verhoeven et al. 2015 [24]	Within subject	43	Seventh grade students exhibiting an inactive lifestyle	12 …16

**Table 3:** Included studies with perceived game experience variables as outcome measures reported by assessment and author

Study	Outcome (perceived game experience variables)	Intervention (game)	Intervention (mode)	Results
Feltz et al. 2012 [34]	1) persistence 2) ratings of perceived exertion (RPE)	EyeToy: Kinetic on PlayStation 2, 5 different plank positions	a) low discrepancy partnered (1:1.01 in persistence) b) moderate discrepancy partnered (1:1.4 in persistence) c) high discrepancy partnered (1:2 in persistence) d) individual control	1) significant gender effect in all conditions (a,b,c,d) in persistence; larger gain in persistence for all partnered conditions (a,b,c) than in the individual controls (d); gain significantly larger in the moderate-discrepancy condition (b) compared to other two partnered conditions (a,c) 2) RPE (ratings of perceived exertion) higher in partnered conditions (a,b,c) compared to individual control (d)
Ganesh et al. 2014 [27]	1) Did you realize what the connection forces were? 2) Did you perform better in the presence or absence of interaction forces? 3) Did you feel fatigue during the experiment?	tracking task	absence of connection forces vs. presence of connection forces appearing as (a,c,d,e,f), control group (b) a) novice-novice interaction (20 participants) b) solo (10) c) force-playback (10) d) trajectory-playback (14) e) expert connect (10) f) target connect (10)	1) in all non-target connected force fields (a,c,d,e) 2 of 54 realized representation of the forces; in target connected force fields (f) all realized representation of the forces 2) in (a,c,d,e) all subjects believed, that they performed worse in presence of forces; (f) all subjects believed, that they performed better in presence of forces 3) no fatigue felt by participants
Goršič et al. 2017 [31]	1) Intrinsic Motivation Inventory subscales interest/enjoyment, perceived competence, effort/importance, pressure/tension 2) overall experience	Pong	a) single-player game b) competitive game c) cooperative game with split field d) cooperative game with shared field	1) competitive game (b) more enjoyable and higher effort/importance than cooperative game with a split filed (c) 1,2) if competitive game (b) was favourite mode then competitive game (b) more enjoyable than single-player game (a)
Goršič et al. 2017 [32]	1) Intrinsic Motivation Inventory subscales interest/enjoyment, perceived competence, effort/importance, pressure/tension 2) preference 3) fun 4) tension 5) duration	Pong Game	a) competition session 1 b) competition session 2 c) competition session 3 d) single-player	home rehabilitation group: 1) effect of session regarding enjoyment/interest and perceived competence, in single-player (d) lower enjoyment/interest than in competition session (a) or (c), conversation level correlated with pressure/tension in competition sessions (a-c) 2) playing with someone else (a-c) more frequently preferred (11, 7 strongly, 4 weakly) than playing alone (d) (4), conversation level in competition session (a) correlated with preference 3) playing with someone else (a-c) was stated being more fun (11, 6 much, 3 moderately, 2 slightly) than playing alone (d) (1 much), conversation level in competition session (a) correlated with fun 4) playing with someone else (a-c) was stated being more tense (8, 3 moderately, 5 slightly) than playing alone (d) (2, 1 moderately, 1 slightly) 5) no difference regarding play duration clinical environment group: 1) effect of session regarding effort/importance 2) playing with someone else (a-c) more frequently preferred (9, 5 strongly, 4 weakly) than playing alone (d) (5, 2 stronly, 3 weakly) 3) playing with someone else (a-c) was stated being more fun (10, 5 much, 5 moderately, 2 slightly) than playing alone (d) (3, 2 much, 1 moderately) 4) playing with someone else (a-c) was stated being more tense (8, 3 moderately, 5 slightly) than playing alone (d) (2, 1 moderately, 1 slightly) 5) competition session 2 (b) longer play duration than competition session 1 (c) and single-player (d)
Goršič et al. 2017 [33]	1) Intrinsic Motivation Inventory subscales interest/enjoyment, perceived competence, effort/importance, pressure/tension 2) preference	Pong game	a) no difficulty adaptation b) manual difficulty adaptation c) automatic difficulty adaptation	uninpaired pairs: 1) manual adaptation (b) higher enjoyment/interest, effort/importance, pressure/tenstion than no adaptation (a); automatic adaptation (c) higher enjoyment/interest, effort/importance, pressure/tension than no adaptation 2) manual adaptation (b) more frequently preferred (18) to no adapation (a) (2), automatic adaptation (c) more frequently preferred (17) to no adaptation (a) (3), manual adaptation (b) more frequently preferred (13) to automatic adaptation (c) (3) impaired-unimpaired friends: 1) no statistical test on Intrinsic Motivatin Inventory 2) automatic adaptation (c) more frequently preferred (4, 3 strongly, 1 weakly) than manual adaptation
Johnson et al. 2008 [28]	1) experience 2) user preference 3) willingness to play	tic-tac-toe	a) single-player PC b) multiplayer robotic with aid of a game camera c) multiplayer robotic with aid of a game camera and audio d) multiplayer robotic with aid of a game camera, a user camera and audio	1) multiplayer robotic (b,c,d) more valuable, more interesting, more collaborative, requiring less effort, more choice, less tensing than single-player PC (a) 2) multiplayer robotic with aid of a game camera, a user camera and audio (d) preferred to multiplayer robotic with aid of a game camera and audio (c) which was preferred to multiplayer robotic with aid of a game camera (b) 3) participants are willing to player longer in the multiplayer robotic conditions (b,c,d) compared to single-player condition (a)
Mace et al. 2017 [29]	1) user preference 2) engagement	BalloonBuddies	a) single-player b) dual-player	healthy-healthy experiment: 1) dual-player (b) was preferred; increased perceived pressure and increased perceived effort in dual-player(b); correlation between perceived competence and performance measures in single-player (a); but not in dual-player (b) 2) enjoyment/interest (IMI) more positively in dual-player(b); no significant difference in perceived competence (IMI) or effort/importance (IMI) patient-expert experiment: 1) dual-player (b) was preferred, increased perceived competence, increased perceived effort and reduced perceived difficulty; correlation between perceived competence and performance measures in single-player (a), but not in dual-player (b) 2) enjoyment/interest (IMI), perceived competence (IMI) and effort/importance (IMI) more positively in dual-player (b)
Novak et al. 2014 [30]	1) experience with last game mode in subsets of intrinsic motivation (interest/enjoyment, perceived competence, effort/importance, pressure/tension) 2) overall game experience	Air hockey	a) single-player b) competitive Interaction c) cooperative Interaction	1) higher motivation in interest/enjoyment for competitive interaction (b) and cooperative interaction (c) compared to single-player (a); higher motivation in perceived competence for single-player (a) and competitive interaction (b) compared to cooperative interaction (c); higher motivation in effort/importance for competitive interaction (b) compared to single-player (b); higher motivation in pressure/tension for competitive interaction (c) compared to single-player (a) and cooperative interaction (c) 2) overall, players liked either the competitive mode (b) but not the cooperative (c) mode or vice versa
Peng and Crouse 2013 [35]	1) enjoyment 2) future game-play motivation	Space Pop mini-game in Kinetic Adventures	a) single-player b) cooperate with friend or stranger; same space c) compete with a friend or stranger; separate spaces	1) less enjoyment in single-player (a) as in cooperative (b) or competitive (c) group; 2) less future game-play motivation in single-player mode (a) as in cooperative (b) or competitive (c) group;
Peng and Hsieh 2012 [36]	1) manipulation check (perception of game mode) 2) motivation 3) goal commitment	Balloon Popping game	a) compete with friend b) compete with stranger c) cooperate with friend d) cooperate with stranger	1) 11 subjects did not pass the manipulation (wrong perception of game mode, e.g. subject perceived competition instead of cooperation) 2) higher motivation and level of effort in cooperative (c,d) compared to competitive mode (a,b);3) higher goal commitment in cooperative (c,d) compared to competitive mode (a,b); playing with a friend (c) resulted in greater goal commitment compared to playing with a stranger (d) in the cooperative goal structure context
Staiano et al. 2012 [25]	1) intrinsic motivation 2) psychological attractiveness of game design	Nintendo Wii Exergame: “The Wii Active game”	a) cooperative interaction b) competitive Interaction	1) favouring of the cooperative (a) over the competitive (b) exergame condition for motivation 2) favouring the cooperative (a) over the competitive (b) exergame condition for ratings of psychological attraction
Staiano et al. 2013 [26]	1) self-efficacy 2) self esteem 3) peer support	Nintendo Wii Active Exergame	a) competitive exergame b) cooperative exergame c) control group (no exergaming)	1) cooperative group (b) increased self-efficacy more than the control group (c); no difference between competitive (a) and cooperative (b) group or competitive (a) and control group (c) 2) no effects in self-esteem 3) cooperative (a) and competitive (b) group increased more in peer support than control group (c)
Verhoeven et al. 2015 [24]	1) game enjoyment	Kinect Sports (boxing, bowling, tennis, baseball, golf), Just Dance 3	a) single-player mode b) two-player mode	1) no difference between two-player mode (b) and single-player mode (a); no sex differences were found; higher game enjoyment for two-player mode (b) in baseball; no correlation between energy expenditure and game enjoyment for most exergames

**Table 4:** Included studies with physical performance as outcome measures reported by assessment and author

Study	Outcome (physical performance)	Intervention (game)	Intervention (mode)	Results
Feltz et al. 2012 [34]	1) heart rate	EyeToy: Kinetic on PlayStation 2, 5 different plank positions	a) low discrepancy partnered (1:1.01 in persistence) b) moderate discrepancy partnered (1:1.4 in persistence) c) high discrepancy partnered (1:2 in persistence) d) individual control	1) higher heart rate in partnered conditions (a, b, c) compared to individual control (d)
Ganesh et al. 2014 [27]	1) mean distance to target 2) learning (reduction of mean distance to target)	tracking task	absence of connection forces vs. presence of connection forces appearing as (a,c,d,e,f), control group (b) a) novice-novice interaction (20 participants) b) solo (10) c) force-playback (10) d) trajectory-playback (14) e) expert connect (10) f) target connect (10)	1) novice-novice interaction (a) improved the task performance whether the partner performance was better or worse than the individual performance; highest improvement with stiffness K=120 N/m compared to K=60 N/m or K=180 N/m; force-playback (c) significantly different to novice-novice interaction (a); trajectory-playback, expert connect and target connect (d,e,f) improved the task performance, but significantly less than novice-novice interaction (a); condition (a,c,d) affects behavior; partner performance affects behaviour; target connect (f) performance improvement less then expert connect(e) 2) novice-novice interaction (a) achieved significantly better performance intermittently interacting with a partner compared to solo (b) condition
Goršič et al. 2017 [31]	1) root-mean-square (RMS) value of hand velocity 2) mean absolute values of hand velocity	Pong	a) single-player game b) competitive game c) cooperative game with split field d) cooperative game with shared field	1) competitive play (b) higher RMS value than in other modes (a,c,d) 2) competitive play (b) higher mean absolute value of hand velocity than in other modes (a,c,d)
Goršič et al. 2017 [32]	1) root-mean-square (RMS) value of velocity	Pong Game	a) competition session 1 b) competition session 2 c) competition session 3 d) single-player	home rehabilitation group: 1) single-player (d) lower RMS than in other modes (a,b,c) clinical environment group: 1) no difference
Goršič et al. 2017 [33]	1) root-mean-square (RMS) of velocity	Pong game	a) no difficulty adaptation b) manual difficulty adaptation c) automatic difficulty adaptation	1) no statistical test
Johnson et al. 2008 [28]	1) game performance 2) total reach displacement 3) movement smoothness 4) peak velocity 5) movement time	tic-tac-toe	a) single-player PC b) multiplayer robotic with aid of a game camera c) multiplayer robotic with aid of a game camera and audio d) multiplayer robotic with aid of a game camera, a user camera and audio	1) game performance influenced motivation 2) no significant difference between multiplayer robotic with aid of a game camera (b) and multiplayer robotic with aid of a game camera, a user camera and audio (d) 3) no significant difference between (b) and (d) 4) no significant difference between (b) and (d)
Mace et al. 2017 [29]	1) score 2) target tracking 3) stability 4) effort 5) smoothness	BalloonBuddies	a) single-player b) dual-player	healthy-healthy experiment: 1) no significant difference 2) accuracy decreased in dual-player condition (b) compared to single-player (a) 3) no significant difference 4) playing with a better partner significantly reduces the effort for the worse performing partners and vice versa for the better partner 5) no significant difference patient-expert experiment: 1-3) patient improved in score, accuracy and stability4-5) little association between patient performance and the effect of dual-player mode (b) on effort or smoothness
Novak et al. 2014 [30]	1) game scores	Air hockey	a) single-player b) competitive Interaction c) cooperative Interaction	1) 19 of the 30 subjects scored higher than the computer in the single-player mode, on average scoring 2.0 points more than the computer in single-player (a); by definition, 15 subjects won and 15 lost in the competitive mode (b), with the mean difference between subjects of 17.7 points; all subjects scored higher than the computer in the cooperative mode (c), with each pair on average winning by 16.3 points
Peng and Crouse 2013 [35]	1) physical exertion	Space Pop mini-game in Kinetic Adventures	a) single-player b) cooperate with friend or stranger; same space c) compete with a friend or stranger; separate spaces	1) significant more physical exertion in single-player (a) than cooperative mode (b); no significant difference between single-player (a) and competitive group (c)
Peng and Hsieh 2012 [36]	1) game performance	Balloon Popping game	a) compete with friend b) compete with stranger c) cooperate with friend d) cooperate with stranger	1) no effect in performance regarding mode (competition a, b versus cooperation c, d) or relationship (friend a, c versus stranger b, d)
Staiano et al. 2012 [25]	1) energy expenditure dependency on subsets of intrinsic motivation (sensory immersion, control/choice, challenge/optimal difficulty, goal setting, and feedback) and psychological attraction	Nintendo Wii Exergame: “The Wii Active game”	a) cooperative interaction b) competitive Interaction	1) high levels of intrinsic motivation due to control/choice predicted high amounts of energy expenditure (a, b); high levels of motivation due to goal setting and sensory immersion predicted lower amounts of energy expenditure; psychological attraction to game play did not significantly predict energy expenditure
Staiano et al. 2013 [26]	1) weight change	Nintendo Wii Active Exergame	a) competitive exergame b) cooperative exergame c) control group (no exergaming)	1) cooperative group (b) lost significantly more weight than the control group (c), competitive group (a) did not significantly differ from the other groups (b, c)
Verhoeven et al. 2015 [24]	1) energy expenditure	Kinect Sports (boxing, bowling, tennis, baseball, golf), Just Dance 3	a) single-player mode b) two-player mode	1) children consumed more energy in a two-player mode (b) than in a single-player mode (a); no sex differences were found; children consumed significantly more energy in a two-player mode (b) when playing boxing, tennis, and dancing and vice versa when bowling

**Table 5:** Included studies with personality factors as outcome measures reported by assessment and author

Study	Outcome (personality factors	Intervention (game)	Intervention (mode)	Results
Goršič et al. 2017 [31]	1) personality	Pong game	a) single-player game b) competitive game c) cooperative game with split field d) cooperative game with shared field	1) interest/enjoyment correlated with agreeableness in single-player game (a) and with competitiveness in cooperative game with split field (c); effort/importance correlated with agreeableness in single-player game (a), in cooperative game with split field (c), and cooperative game with shared field (d); effort/importance correlated intellect/imagination in single-player game (a), in the cooperative games (c) and (d); perceived competence correlated with competitiveness in cooperative game with split field (c)
Goršič et al. 2017 [32]	1) personality	Pong Game	a) competition session 1 b) competition session 2 c) competition session 3 d) single-player	home rehabilitation group: 1) conscientiousness correlated with preference and fun; conscientiousness and emotional stability correlated with how often participants play computer games; agreeableness was correlated with how often participants play computer games clinical rehabilitation group: 1) agreeableness correlated with how often participants play computer games
Novak et al. 2014 [30]	1) personality	Air hockey	a) single-player b) competitive Interaction c) cooperative Interaction	1) most commonly selected inputs to the classifier predicting mode preference were emotional stability, competitiveness, and the co-player’s extraversion

Perceived game experience is discussed as the expressed opinions gathered from the activity. The opinion was assessed either during or after the task and either by answering a questionnaire or by assessing performance attributes (e.g. player’s decision on duration of task performance). Physical fitness is considered as a set of attributes that people have or achieve to perform a physical activity as defined by Caspersen [18]. Physical performance is considered as the performed physical activity represented by the amount of useful work accomplished within the task. Assessment of the usefulness of the work is defined by the task designers.

Because we expected the interventions and reported outcome measures to be markedly varied, we focused on a description of the studies and their results, and on thematic synthesis as defined by Thomas and Barnett-Page [19, 20].

### Assessment of study quality

Our critical appraisal of the studies was based on a checklist designed for assessing the methodological quality of both randomised and non-randomised studies of healthcare interventions [21]. The checklist assesses biases related to reporting, external validity, internal validity and power. Six items were not considered in this review: adverse effects (item 8), follow-up analyses (items 9 and 26), representativeness of treatment locations and facilities (item 13), allocation concealment for participants (item 14), and blinding of investigators (item 15). These items were excluded because there were not any follow-up studies available that considered the given type of intervention (items 9 and 26), or because we considered these items as being of minor significance for this review (items 8, 13, 14 and 15).

The remaining 21 items were applied by two reviewers (KB, AS) to assess the methodological quality of the selected full text studies (Additional file 2). The total possible score was 22 points, whereas every item can be scored 0 or 1 point except for the confounding description (item 5; 0, 1 or 2 points). Scoring for consistency in recruitment time period (item 22) was, compared to the original checklist, changed to scoring for stating time period of intervention. Scoring for statistical power (item 27) was simplified to a choice between 0 or 1 points depending on the level of ability to detect a clinically important effect. The scale ranged from insufficient (*β*<70*%*=0points) to sufficient (*β*≥70*%*=1point). To assess the level of agreement between the investigators a Cohen’s kappa analysis was performed on all items on the checklist. In accordance with Landis and Koch’s benchmarks for assessing the agreement between raters a kappa-score of 0.81...1.0 was considered almost perfect, 0.61...0.8 was substantial, 0.41...0.6 was moderate, 0.21...0.4 was fair, 0.0...0.2 were slight and scores <0 were poor [22]. The PRISMA-statement was followed for reporting items of this systematic review [23]. Therefore, eligible criteria, information sources, and search strategy were defined pre-search (30.10.2014) and remained unchanged (Additional file 1). Study selection, data collection, and data reporting are fully described within this paper. However, the systematic review is not registered in any data base.

## Results

### Study selection

The search provided a total of 1889 references. After adjusting for duplicates, 1330 remained. Of these, 1304 were discarded because they were out of scope of this review. The remaining 26 potentially relevant articles were supplemented by 7 additional references retrieved by a manual search. This resulted in a total of 33 articles eligible for full-text reading. After full-text reading, 19 articles were excluded because neither an intervention nor social interaction was presented (Fig. 1). Thirteen articles were finally reviewed (Table 2).

### Study characteristics

All studies were published in English. The publication dates ranged from 2008 to 2017. In the selected studies, participants were seventh-graders [24], high-school students [25, 26], adults [27–30], arm impaired [29–33], and undergraduate students [33–36]. The age of the participants ranged from 12 years to over 90 years whereas four publications did not include full information about minimal and maximal age [29, 31, 33, 34].

### Devices, games and multiplayer modes

Six studies used a commercially available exergaming input device, such as a Nintendo Wii controller or a Kinect system, in combination with a commercially available game [24–26, 35], or an in-house developed game [34, 36]. Five studies used a haptic manipulator or arm rehabilitation system with an in-house developed game [28, 30–33]. One study used a grip transducer with an in-house developed game [29].

In six studies addressing multiplayer modes, a competitive mode was compared to a cooperative mode [25, 26, 30, 31, 35, 36]. Four of these studies also compared competitive or cooperative gaming against a single-player mode [26, 30, 31, 35]. Six studies compared either competitive or cooperative gaming against a single-player mode [24, 27–29, 32, 34]. Only one study compared a competitive mode to a single-player mode in different games [24] and one study compared multiple sessions of a competitive mode to a single-player mode [32]. One study investigated the influence of external forces, including forces from partner players, on performance and motor learning [27]. Included studies also compared discrepancy levels of a participant’s game performance to a competitor’s game performance [34], and different methods to decrease discrepancy in a multiplayer rehabilitation game [33]. Furthermore, included studies compared friend- and stranger-paired group multiplayer gaming [35], gaming in home and clinical environments [32], and different levels of interaction in a tele-rehabilitation game environment [34].

### Assessment

#### Assessment of perceived game experience

Eleven out of thirteen articles assessed the effect of the intervention on the motivation of the player (Table 3). To measure the players’ motivation, the Intrinsic Motivation Inventory (interest/enjoyment, perceived competence, effort/importance, pressure/ tension) [37] was used seven times [28–33, 36], Malone’s theory of intrinsic motivations for learning (sensory immersion, control/choice, challenge/optimal difficulty, goal setting and feedback) [38] was used once [25], the Kids Game Experience Questionnaire [39] was used once [24], and an evaluation of motivation related adjectives (boring (reverse-coded), exciting, enjoyable, entertaining, fun, interesting, pleasant) [40] was used once [35].

A ranking regarding preferred game mode based on subsets of the Intrinsic Motivation Inventory (interest/enjoyment, perceived competence, effort/importance, pressure/tension) [37] was used in four studies [28–31] and a weighted preference in one study [33].

Assessment of goal commitment [41, 42] was used once [36], and assessment of psychosocial attractiveness of game design regarding interpersonal communication in the four factors social interaction, collaboration, individual feedback and team feedback [43] once [25].

The Koehler effect [44], assessed by measuring the difference in persistence in executing the intervention task, was used once [34].

Psychosocial factors such as self-efficacy [45], efficacy self-esteem [46] and peer support [47] were assessed in one study [26].

Intervention specific questions for perception assessment of the external forces and fatigue were used in one study [27].

#### Assessment of physical performance

Five out of thirteen articles assessed physical fitness by calculating energy expenditure (Table 4): weight difference by measuring weight before and after the intervention [26], comparing the heart rate during an intervention with heart rate during individual control condition [34], calculating the metabolic equivalent of task [24] as defined by Ainsworth [48], and averaging the acceleration profile assessed during the intervention [25, 35] as introduced by Puyau [49].

Five out of thirteen studies, [27–30, 36], assessed physical performance by comparing game performance scores. Three out of thirteen studies, [31–33], used the root-mean-square (RMS) of the velocity profile of the hand movement (introduced by Van Der Pas [50]); one out of these three studies also reported mean velocity [31]. One study, [28], assessed physical performance by extracting the peak velocity of the hand movement. One study, [34], assessed perceived exertion with the Borg scale [51].

#### Assessment of personality factors

In three studies [30–32], assessments of perceived game experience and physical performance were compared with assessments of personality as suggested by Goldberg [52] (Table 5).

### Influence of multiplayer games on game experience and performance

**Fig. 2:**
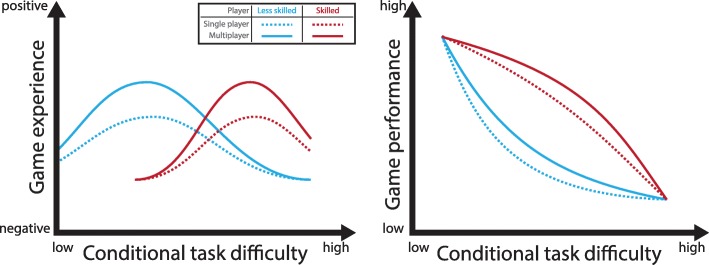
Summary of the review. Multiplayer game modes (solid lines) have been shown to differ in their impact compared to single-player game modes (dotted lines) in health-related applications for two differently skilled players (dark red, light blue). In comparison to single-player modes (dotted lines), multiplayer game modes (solid lines) have been shown to positively influence both game experience (left) and game performance (right). The benefit of multiplayer modes is present for players of all skill levels (light blue representing less skilled players, dark red representing skilled players, respectively) and at all conditional task difficulties

The majority of the reviewed studies state that social interaction through multiplayer game settings improve both game experience and game performance (Fig. 2). All studies that examined game experience stated that their results proved that multiplayer modes positively influenced game experience [24, 26, 28–32, 34, 35]. Most studies stated that multiplayer games led to better game performances or higher physical exertion in most of the measured dimensions [24, 26, 27, 29, 31, 34]. Two studies stated that single-player mode improved game performance when compared to multiplayer modes or increased physical exertion in certain dimensions [29, 35]. Two studies also found correlation of game experience and game performance [25, 28].

### Quality evaluation

The agreement on study quality between the two reviewers was almost perfect. The estimated Kappa value was 0.99 with a confidence interval ranging between 0.96 and 1.00 (*α* = 0.05). The percentage of agreement between the two reviewers was 99.3%. The mean quality score was 12.1 points (maximum: 22 points, range: 10-14 points), the median value was 12 points and the mode was 11 points. The mean score for reporting was 6.2 points (maximum: 9 points; range: 5-8 points), for external validity 0.8 points (maximum: 2 points; range: 0-2 points), for internal validity (bias) 3.9 points (maximum: 5 points; range: 3-4 points), for internal validity (confounding) 1.2 points (maximum: 5 points; range: 0-2 points) and for power 0.0 points (maximum 1 point; 0 points), see Additional file 2.

## Discussion

### Study selection

Our search resulted in thirteen articles fulfilling the inclusion criteria. These articles evaluated the psychological and physiological experience of multiplayer gaming using various forms of player interaction in studies on neuromuscular patients, overweight adolescents, and students. The thirteen articles remain small compared to 1031 papers related to “robot-assisted training” and to 444 papers related to “exergaming” that were published until July 2018 [53]. This highlights that multiplayer gaming seems not yet systematically considered in neuromuscular therapy or other health-related training. The combination of both the small number of included papers and the heterogeneity in their methods, prevents us from giving a clear answer to the research question.

### Devices and games inconsistently discussed

The selection process of devices and of the commercially available games was consistently not discussed within any of the thirteen articles. The design process of the in-house developed games was discussed in three articles [27, 29, 34]. Feltz et al. [34] and Ganesh et al. [27] designed the games according to the paradigm to be tested, i.e., Koehler effect [44] and reactive motor adaptation, respectively. Mace et al. [29] explained the game design process, and how their multiplayer game mode explicitly demands a collaborative behavior [7]. To better discuss and compare the study results, the targeted behavior and the actual game modes selected or designed, should generally be reported in more detail.

### Limited diversity of multiplayer modes

**Fig. 3:**
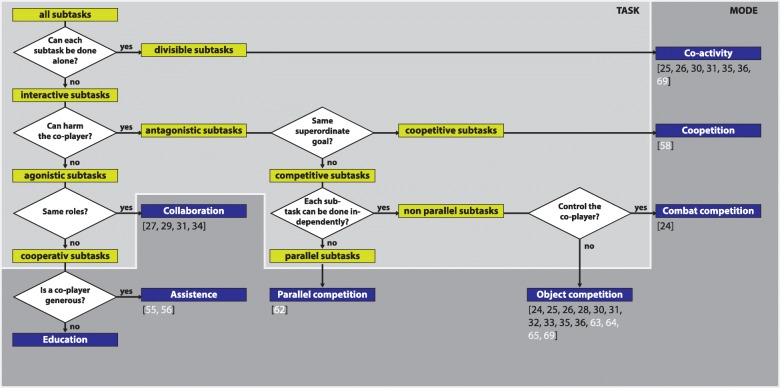
Determination of multiplayer modes. According to the taxonomies of Jarrassé [7], Konert [59], and Mueller [58], the applied multiplayer mode can be determined based on the task characteristics. The task characteristics and the players’ behavior define the behavioral characteristics of the multiplayer mode. The references of the included studies (black) and feasibility studies (white) are placed at the multiplayer modes applied in the corresponding studies. The full variety of multiplayer modes was not covered by the included studies and therefore needs further investigation

The multiplayer modes in the reviewed studies have been commonly named competitive, collaborative and cooperative mode. According to the taxonomy proposed by Jarrassé et al. [7] and others (Fig. 3), some modes of training in the reviewed studies should be termed co-active (a task that can be solved by individual player) rather than cooperative (playing in the same team with different roles according to own individual skills, thus, a role being either “supported” or “supportive”; see also Sawers and Ting [54]). Although a cooperative mode in that sense has not been considered in the reviewed studies, this mode may have great potential in robot-assisted rehabilitation systems since the roles of “supported” and “supportive” are often given in a therapeutic setting with patient and therapist [55, 56]. The collaborative mode (playing in the same team with equal roles) was used in four studies [27, 29, 31, 34]. Differences in effects of collaberative and competitive modes in health-promoting exergames have already been discussed by Marker and Staiano [57].

The distinction of competitive modes, as done by Mueller [58], seems reasonable to predict training outcome. For instance, in combat gameplay, physical effort was higher than in object competition when both players controlled the same object [24].

Coopetition, i.e., competition and cooperation in combination, has been proposed as a new mode of multiplayer gaming [59]. Derived from a business concept, coopetition can be linked to a social health behavior where people compare their health behavior with others sharing the same health-related goal [59, 60]. Platforms such as social media enable this health behavior. This “coopetitive” behavior can be targeted by games that monitor the health progress in an understandable and comparable representation, e.g. weight loss. Robot-assisted neurorehabilitation can provide similar representations using parameters such as range-of-motion assessments and performance measures in reaching tasks [61].

### The potential of playing with friends and relatives as co-players

Another example to combine competition and cooperation has been tested in a game-based learning study [62]. In this study, a second level of social interaction was introduced. In the first level of interaction, the teams solved subtasks together in a cooperative manner. In the second level of interaction, the teams interacted with other teams in a competitive manner. Such combinations of competition and cooperation are applicable in clinical environments. Subgroups of patients in clinics or different clinics could compete against each other. Part of the competition could be a comparison of therapy progress, duration of device usage, or virtual points in rehabilitation games. The groups of patients can motivate their teammates to cooperatively contribute to their team’s score by training more. Such a setup is possible in telerehabilitation since participating in remote places does not seem to reduce the motivation to compete: game experience and game score have been shown not to be affected when competing in different rooms compared to cooperating in the same room [35].

Cooperating in a team might be even more motivating when the team member is a friend or a relative. Playing with a friend in a cooperative mode resulted in greater goal commitment compared to playing with a stranger [36]. Regarding rehabilitation games, this implies that in a cooperative game, a well-known game partner – such as a family member – may be preferred over a lesser-known patient or therapist. For competitive modes it was already shown that playing with well-known game partners does improve the game experience when compared to playing with lesser known game partners [31]. Such game play requires input devices for different skill levels, e.g. rehabilitation robots for a severely impaired patient and exergaming devices, such as a Nintendo Wii, for moderately impaired and unimpaired players. Tasks solved by players with different skill levels were discussed in the concepts of flow and of the challenge point framework [9, 16]. However, both concepts are limitedly discussed with regard to multiplayer games using different input devices for different skill levels.

### Balancing multiplayer interventions in the context of the flow model and the challenge point framework

The flow model considers the immediate task difficulty and current skill level, thus, defines whether the player is experiencing boredom, flow or frustration. We use this model as a basis for a description of a player’s specific skill level: the game experience profile. The inverted-U-shape dependency of game experience on task difficulty has been confirmed in multiplayer health-related games [34]. The global maximum of the game experience profile represents the point of highest rated game experience. Flow is expected to be experienced close to this point.

Different game modes result in differently shaped game experience profiles. These profiles are influenced by both the game mode and the players’ skills in the game, thus, resulting in different maxima. According to the flow model, the game experience of skilled players demands higher task difficulty to achieve maximum game experience [9]. In studies applying multiplayer modes, a cooperative mode can result in a better game experience compared to a competitive mode [36]. This increase in game experience could be represented by an overall shift upwards in the game experience profile or a game experience profile with less declination; both represented by a widened flow zone — a hypothesis that has yet to be confirmed. Novak et al. [30] found that the player’s preferred mode may be predicted based on a correlation between the player’s preferred multiplayer mode and personality. Therefore, a player-tailored selection of multiplayer mode may result in a better game experience, too.

The challenge point framework, introduced by Guadagnoli [16], provides a theoretical basis to conceptualize the effects of task difficulty in motor learning. In competitive games, the game experience is linked to the skill level of the opponent [9]. A discrepancy in skill level makes the weaker player feel frustrated and the better player bored. Changing the conditions by adapting visual, auditory or haptic game elements can reduce the differences in skill level between players. The terminology of the challenge point framework can be used to describe the difficulty of any task, not only regarding the conditions, but also the characteristics of the task and the skill level of the subject.

### Conditional task difficulty: complementing the terminology

The challenge point framework defined the terms **nominal task difficulty**, i.e. the characteristics of the task only, and the **functional task difficulty**, i.e. the difficulty of the task relative to the skill level of the player and the conditions under which the task is performed. However, the term functional task difficulty does not allow for a distinction of the skill level of the player and the conditions under which the task is performed. Therefore, we propose an extended definition by introducing the term **conditional task difficulty**, i.e. the difficulty of the task relative to the conditions under which the task is performed. This extended definition is particularly relevant for environments where the conditions of the task can be adapted online, as when using haptic robots that can adapt the support of a patient based on his/her performance. In addition, this extended definition allows to collectively report task difficulty when a game is played with different input devices by the involved players.

### Changing the individual conditions to optimize game experience

By changing the condition individually, we can change the players’ game performance in relation to their individual skill level. In two included studies and five exemplary feasibility studies, game design features such as speed, frequency of actions, or avatar size were adapted in single and multiplayer rehabilitation games to account for differences in skill level [32, 33, 63–67]. Such design features may change the conditions generally for both players, instead of for each player individually. In addition, these features may visually reveal the different skill levels of the players which can be embarrassing for the worse performing player.

**Fig. 4:**
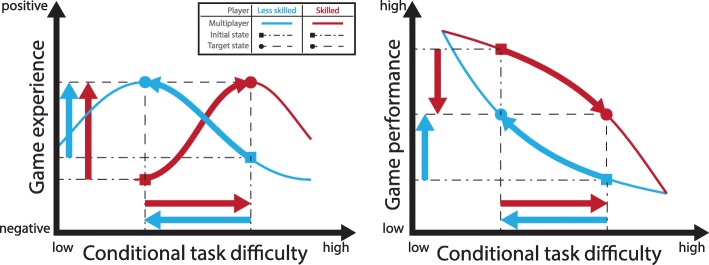
Difficulty adaptation based on individual condition setting in multiplayer games. Game experience (left) can be optimized by balancing the game performance (right). – Left: The initial game experience under nominal conditions relates to the skill level of the opponent and is non-optimal for differently skilled players (squares). Optimal game experience is perceived by the players when the condition adapts the difficulty towards the players’ skill level (circles). – Right: A common initial game performance state consists of a conditional task difficulty and its corresponding player specific game performance (square). Player specific difficulty adaptation can balance the game performances of the two players (circles)

Robotic devices offer unique design features to tailor the condition to the players’ motor abilities [68]. For instance, haptic force fields have been used to adjust the task difficulty of a training task. These force fields could be used in multiplayer games to individually set the level of difficulty [69]. In all difficulty adapting strategies, we have to consider that a change in condition for one player implicitly introduces a change in condition for the other one, since the players are connected over the nominal task itself. If we support the less skilled player, or hinder the more skilled player, in succeeding in the task we approach the highest game experience for both players (Fig. 4).

Performance-balanced games improved game experience compared to non-balanced games in one of the included studies and several feasibility studies [33, 63, 64, 69]. In therapy settings where the level of difficulty cannot be individually adapted, collaborative game designs may be preferred since they may require less similarity in skill level to achieve flow [29].

The motivation in cooperative modes can even be increased with conjunctive tasks design (i.e. collaborative according to Jarrassé [7]) facilitating the Koehler effect [70]. The Koehler effect occurs when an inferior team member performs a difficult task better in a team than one would expect from knowledge of his or her individual performance. The effect has been found to be strongest in conjunctive task conditions in which the group’s potential productivity towards a cooperative goal is equal to the productivity of its least capable member [71]. Experience with the Koehler effect implies that multiplayer difficulty adaptation targeting a moderate discrepancy compared to the player’s performance can optimise the game experience of the participants [27, 34]. Such small discrepancies can be achieved by robotic devices as they can individually assist or challenge players [15].

### Different assessments hinder inter-study comparison

#### Assessment of perceived game experience

In general, measures of game experience can be acquired using physiological measures and questionnaires; only the latter were present in the included papers. Physiological measures, e.g. sensors measuring cardiac activity, significantly improve the assessment of game experience [72]. However, the required additional set of sensors may disturb game play and effort by the study operators. Among the presented questionnaires, the Intrinsic Motivation Inventory is widely used in various sports interventions and can therefore allow inter-study comparison [37]. However, the Intrinsic Motivation Inventory does not necessarily have good discriminative power as pointed out by one study [30]. That is why the participants have been asked to also rank the games regarding game experience related questions (e.g. “Which game mode do you prefer?”).

Inventories regarding psychosocial attractiveness of game design in the dimensions of social interaction, collaboration, individual feedback and team feedback, can extend motivation inventories when different multiplayer modes are compared [25]. The perceived multiplayer mode, e.g. playing in a cooperative or competitive mode, will influence the answers regarding psychosocial attractiveness. However, the multiplayer mode might not be perceivable to all players. Thus, should be checked after each mode if the mode is not introduced accordingly [36].

The benefit of social interaction could be increased by integrating visual, auditory/verbal, and haptic elements. In the cognitive task study of Yu et al. [62] conversely, hiding of the opponent team (anonymous opponents) in a competitive mode showed improved game experience and satisfaction compared to visual presence of the opponent team. Hence, visual presence of the opponent seems to negatively influence game experience. In contrast, Johnson et al. [28] stated that the more modalities of social interaction integrated in motor tasks, the more enjoyable for the players. The perceived intensity of social interaction in different modalities seems to vary between task and player. In the study of Yu et al. [62] the intensity of social interaction within the team members might superpose any interaction with the opponent team. Therefore, assessing the perceived intensity of social interaction provided by different modalities and involved people is suggested. In the study of Gorsic et al. [32], the level of verbal interaction was assessed by a study operator. However, a standardized assessment for perception of interaction intensity has not been established yet.

#### Assessment of physical performance

Energy expenditure is a common measure for physical fitness regarding physical performance [73]. Various methods to measure energy expenditure or their consequences include: the determination of acceleration profiles [25, 35], feature extraction of the velocity profile [28, 31, 33], measuring the weight drop [26], and measuring the heart rate [34]. Alternatively, the Borg scale, used as a measure of perceived physical performance in one study, is a widely accepted and valid measure of exercise intensity [34, 51, 74]. Both methods presented allow inter-game comparison or even comparisons regarding physical performance with non-gaming interventions.

Physical performance corresponds to training intensity and, therefore, predicts improvement in motor function, too [75]. However, neuromuscular therapy and movement training in general are related to motor function and execution and rather than to physical exertion only. Consequently, measures of physical fitness may not be sufficient to provide all relevant information regarding progress in physical functioning. Therefore, game performance score or gamified assessments based on conventional motor functional assessments (e.g. Fugl-Meyer [76]) should be included to complement the evaluation of the influence of the game on physical functioning progress [61].

In games where the end effector of the input device controls the avatar, the end effector position affects the success or failure within the game task. Assessing the end effector position and its impact on task success does not demand functional movement patterns in arm joints. However, functional movements are important in upper limb training. In exoskeleton devices used for rehabilitation training it is possible to measure the movements of non-game controlling joints [77]. One solution is the assessment of quality of movements to measure the player’s functional physical performance. Assessments that are independent of task dimensions do not need a developer’s definition such as the spectral arc length metric [78]. However, the validity of movement quality assessments is under discussion [79].

#### Limitations

We used a structured protocol to guide our search strategy, study selection, extraction of data and statistical analysis. However, limitations of this review should be noted: a publication bias may be present, as well as a language bias, given that we considered only interventions described in published studies and restricted our search to English and German language publications. A bias regarding research fields was generated since we mainly focused on neuromuscular therapy and health-related training.

## Conclusions

Multiplayer modes can enhance the players’ perceived game experience and positively influence the players’ performance. Based on the small number of relevant studies published so far, a conclusion cannot yet be drawn about which multiplayer mode is best during neurorehabilitation training. A meta-analysis of game experience and game performance outcomes may be suggested as soon as more multiplayer studies with homogeneous outcome measures will be published. Nevertheless, this review demonstrated that the players’ individual skill levels and personalities, as well as their role in the game, must be taken into account when selecting and designing multiplayer modes.

Based on the model of flow and the challenge point framework, we suggest an individual adaptation of game conditions, i.e. conditional task difficulty, to assimilate differently skilled players for an optimal game experience. Furthermore, player specific selection of multiplayer modes may result in more robust interventions regarding game experience and requires less assimilation of differently skilled players.

We suggest breaking the limited variety in multiplayer modes and fully exploring multiplayer modes and co-player’s characteristics such as the co-players presence, skill level, personality and relation to the player. We further suggest that future studies use a more stringent research design in which participants are allocated to either single play or multiplayer modes of exercise through randomised assignment.

## Additional files

Additional file 1 Search strategy. (DOC 31 kb)

Additional file 2 Assessment of methodological quality. (DOC 78 kb)
