# Supplementary material for: Trends in robot-assisted and virtual reality-assisted neuromuscular therapy: a systematic review of health-related multiplayer games
Source: J Neuroeng Rehabil. 2018 Nov 19;15:107. doi: 10.1186/s12984-018-0449-9 (PMC6245892; doi:10.1186/s12984-018-0449-9)
Supplement: Supplementary file 1 — Search strategy. (DOC 31 kb) [file 12984_2018_449_MOESM1_ESM.doc]

**Additional File 1 – Search strategy**

| **Area** | **Search terms** |
| --- | --- |
|  |  |
| ***Exergame Therapy*** | (((('virtual reality'/exp OR 'computer interface'/exp OR 'robotics'/exp) AND ('game'/exp OR gam*:ab,ti OR play*:ab,ti)) OR ((video OR comput* OR virtual*) NEAR/3 (gam* OR play* OR train*)):ab,ti AND (therap*:ab,ti OR treat*:ab,ti OR rehab*:ab,ti)) OR exergame:ab,ti OR ((robotics OR 'robot assisted' OR 'robot assistance' OR 'game based' OR ipad) NEAR/6 (train* OR exerc* OR therap* OR treat* OR rehab*)):ab,ti) |
| ***Motivation*** | ('patient attitude'/exp OR 'motivation'/exp OR ((compl* OR adhere* OR progress* OR success* OR succeed*) NEAR/3 (patient* OR therap* OR treat*)):ab,ti OR ((persua* OR motivat* OR engage* OR satisf* OR attitude OR participat* OR preference) NEAR/15 (patient* OR person* OR adult* OR child*)):ab,ti OR ((persua* OR compli* OR motivat* OR engage* OR effort* OR adhere*) NEAR/15 (therap* OR treat* OR rehab*)):ab,ti OR ((break* OR drop* OR stop* OR cancel* OR cut OR fail* OR refus*) NEAR/6 (therap* OR treat* OR rehab*)):ab,ti OR dropout*:ab,ti OR 'drop out':ab,ti OR 'drop outs':ab,ti OR (drop* NEAR/3 out):ab,ti) * |
| ***Multiplayer*** | ('social behavior'/exp OR collaborat*:ab,ti OR cooperat*:ab,ti OR compet*:ab,ti OR 'two player':ab,ti OR multi‐player:ab,ti OR dyad*:ab,ti OR commun*:ab,ti OR alliance:ab,ti OR companion*:ab,ti OR collectiv*:ab,ti OR social*:ab,ti OR interact*:ab,ti) |
