# Supplementary material for: Trends in robot-assisted and virtual reality-assisted neuromuscular therapy: a systematic review of health-related multiplayer games
Source: J Neuroeng Rehabil. 2018 Nov 19;15:107. doi: 10.1186/s12984-018-0449-9 (PMC6245892; doi:10.1186/s12984-018-0449-9)
Supplement: Supplementary file 2 — Assessment of methodological quality. (DOC 78 kb) [file 12984_2018_449_MOESM2_ESM.doc]

**Additional File 2: Assessment of methodological quality**

|  | **Reporting** | | | | | | | |  | **External validity** | |  | **Internal validity (bias)** | | | | |  | **Internal validity (confounding)** | | | | |  |  |  |
| --- | --- | --- | --- | --- | --- | --- | --- | --- | --- | --- | --- | --- | --- | --- | --- | --- | --- | --- | --- | --- | --- | --- | --- | --- | --- | --- |
| Study | Hypothesis/aim/objective clearly described | Main outcomes clearly described in the introduction or methods section | Participants’ characteristics clearly described | Intervention clearly described | Distribution of principal confounders clearly described (max: 2) | Main findings of the study clearly described | Estimation of random variability for main outcomes provided | Actual probability values reported (e.g. 0.035 rather than <0.05) | **Total** (max: 9) | Subjects asked to participate representative for the entire population from which they were derived | Subjects prepared to participate representative of the entire population from which they were recruited | **Total** (max 2) | No retrospective unplanned subgroup analyses | Time period between the intervention and outcome the same for cases and controls | Statistical tests used to assess the main outcomes appropriate | Compliance with intervention/s reliable | Main outcome measures accurate (valid and reliable) | **Total** (max: 5) | Patients in different intervention groups | Patients recruited over the same period of time | Study subjects randomized to intervention groups | Randomised intervention assignment concealed | Adequate adjustment for confounding | **Total** (max: 5) | **Power** (max: 1) | **Total**  **score**  (max: 22) |
| Feltz et al. 2012 [23] | 1.0 | 1.0 | 0.0 | 1.0 | 0.0 | 1.0 | 0.0 | 1.0 | **5.0** | 0.0 | 0.0 | **0.0** | 1.0 | 0.0 | 1.0 | 1.0 | 1.0 | **4.0** | 1.0 | 0.0 | 0.0 | 0.0 | 0.0 | **1.0** | **0.0** | **10.0** |
| Ganesh et al. 2014 [24] | 1.0 | 0.5 | 0.0 | 1.0 | 0.0 | 1.0 | 1.0 | 1.0 | **5.5** | 1.0 | 0.0 | **1.0** | 1.0 | 0.0 | 1.0 | 1.0 | 1.0 | **4.0** | 0.0 | 0.0 | 0.0 | 0.0 | 0.0 | **0.0** | **0.0** | **10.5** |
| Goršič et al. 2017 [25] | 1.0 | 1.0 | 1.0 | 1.0 | 0.0 | 1.0 | 1.0 | 1.0 | **7.0** | 1.0 | 0.0 | **1.0** | 1.0 | 0.0 | 1.0 | 1.0 | 1.0 | **4.0** | 1.0 | 1.0 | 0.0 | 0.0 | 0.0 | **2.0** | **0.0** | **14.0** |
| Goršič et al. 2017 [26] | 1.0 | 1.0 | 1.0 | 1.0 | 0.0 | 1.0 | 1.0 | 1.0 | **7.0** | 1.0 | 0.0 | **1.0** | 1.0 | 0.0 | 1.0 | 1.0 | 1.0 | **4.0** | 1.0 | 1.0 | 0.0 | 0.0 | 0.0 | **2.0** | **0.0** | **14.0** |
| Goršič et al. 2017 [27] | 1.0 | 1.0 | 1.0 | 1.0 | 0.0 | 1.0 | 1.0 | 1.0 | **7.0** | 1.0 | 0.0 | **1.0** | 1.0 | 0.0 | 1.0 | 1.0 | 1.0 | **4.0** | 1.0 | 0.0 | 0.0 | 0.0 | 0.0 | **1.0** | **0.0** | **13.0** |
| Johnson et al. 2008 [28] | 1.0 | 1.0 | 0.0 | 1.0 | 0.0 | 1.0 | 1.0 | 1.0 | **6.0** | 0.0 | 0.0 | **0.0** | 1.0 | 0.0 | 1.0 | 1.0 | 1.0 | **4.0** | 1.0 | 0.0 | 0.0 | 0.0 | 0.0 | **1.0** | **0.0** | **11.0** |
| Mace et al. 2017 [29] | 1.0 | 1.0 | 1.0 | 1.0 | 0.0 | 1.0 | 1.0 | 1.0 | **7.0** | 0.0 | 0.0 | **0.0** | 1.0 | 0.0 | 1.0 | 1.0 | 1.0 | **4.0** | 1.0 | 0.0 | 0.0 | 0.0 | 0.0 | **1.0** | **0.0** | **12.0** |
| Novak et al. 2014 [30] | 1.0 | 1.0 | 1.0 | 1.0 | 1.0 | 1.0 | 1.0 | 1.0 | **8.0** | 1.0 | 0.0 | **1.0** | 1.0 | 0.0 | 1.0 | 1.0 | 1.0 | **4.0** | 1.0 | 0.0 | 0.0 | 0.0 | 0.0 | **1.0** | **0.0** | **14.0** |
| Peng et al. 2012 [31] | 1.0 | 1.0 | 0.0 | 1.0 | 0.0 | 0.0 | 1.0 | 1.0 | **5.0** | 1.0 | 0.0 | **1.0** | 1.0 | 0.0 | 1.0 | 1.0 | 1.0 | **4.0** | 1.0 | 0.0 | 0.0 | 0.0 | 0.0 | **1.0** | **0.0** | **11.0** |
| Peng et al. 2013 [32] | 1.0 | 1.0 | 0.0 | 1.0 | 0.0 | 1.0 | 1.0 | 1.0 | **6.0** | 1.0 | 0.0 | **1.0** | 1.0 | 0.0 | 1.0 | 1.0 | 1.0 | **4.0** | 1.0 | 0.0 | 0.0 | 0.0 | 0.0 | **1.0** | **0.0** | **12.0** |
| Staiano et al. 2012 [33] | 1.0 | 1.0 | 0.0 | 1.0 | 0.0 | 0.0 | 1.0 | 1.0 | **5.0** | 1.0 | 0.0 | **1.0** | 1.0 | 0.0 | 1.0 | 1.0 | 1.0 | **4.0** | 1.0 | 0.0 | 0.0 | 0.0 | 0.0 | **1.0** | **0.0** | **11.0** |
| Staiano et al. 2013 [34] | 1.0 | 1.0 | 0.0 | 1.0 | 0.0 | 1.0 | 1.0 | 1.0 | **6.0** | 1.0 | 0.0 | **1.0** | 1.0 | 0.0 | 1.0 | 0.0 | 1.0 | **3.0** | 1.0 | 0.0 | 0.0 | 1.0 | 0.0 | **2.0** | **0.0** | **12.0** |
| Verhoeven et al. 2015 [35] | 1.0 | 1.0 | 1.0 | 1.0 | 0.0 | 1.0 | 1.0 | 0.0 | **6.0** | 1.0 | 1.0 | **2.0** | 1.0 | 0.0 | 1.0 | 0.5 | 1.0 | **3.5** | 1.0 | 0.0 | 0.0 | 0.0 | 0.0 | **1.0** | **0.0** | **12.5** |
